# Factors influencing effective decrease of controlled attenuation parameters in metabolic-associated steatotic liver disease: A multilevel linear regression analysis at Vajira Hospital

**DOI:** 10.1371/journal.pone.0336294

**Published:** 2025-12-03

**Authors:** Sonsawan Sangprasert, Ratchaneewan Kwancharoen, Witchakorn Ruamtawee, Supatsri Sethasine

**Affiliations:** 1 Division of Gastroenterology and Hepatology, Department of Medicine, Faculty of Medicine Vajira Hospital, Navamindradhiraj University, Dusit, Bangkok, Thailand; 2 Division of Endocrinology, Department of Medicine, Faculty of Medicine Vajira Hospital, Navamindradhiraj University, Dusit, Bangkok, Thailand; 3 Faculty of Medicine Vajira Hospital, Clinical Research Center, Research Facilitation Division, Navamindradhiraj University, Dusit, Bangkok, Thailand; Instituto Nacional de Ciencias Medicas y Nutricion Salvador Zubiran, MEXICO

## Abstract

**Backgrounds:**

Metabolic-associated steatotic liver disease (MASLD) is a growing global health concern. Although several studies have demonstrated associations between baseline metabolic factors and hepatic steatosis, the quantitative influence of these characteristics on the extent of liver fat reduction following lifestyle modification remains unclear. This study aims to analyse the relationship between baseline factors and the modulation of controlled attenuation parameter (CAP) from baseline to 6 months and compare the mean difference in CAP changes of individuals at a telemedicine-based clinic.

**Methods:**

A cohort of MASLD who had hepatic steatosis (CAP ≥ 215 dB/m) with metabolic risk were enrolled. (30 August 2023–30 April 2024). Baseline characteristics, diet and exercise were collected. Multivariable multilevel random intercepts and slope linear regression models were used to analyse the mean difference in CAP change over time for each characteristic adjusted for other variables in the model.

**Results:**

The mean age was 46.93 years, 70% were females. The baseline CAP value was 319.13 ± 42.33 dB/m. Individual baseline age ≥ 45 (−44.52 dB/m [CI −84.86 to −4.17], p = 0.031); higher waist circumference (−87.85 dB/m [CI −153.23 to −22.47], p = 0.008); and a lower BMI (−78.31 dB/m [CI −139.94 to −16.67], p = 0.013) were associated with greater reductions in the mean difference of CAP change. Notably, participants with diabetes (−61.31 dB/m [CI −100.25 to −22.36], p = 0.002) and better glycemic control (−43.49 dB/m [CI −74.00 to −12.99], p = 0.005) exhibited greater liver fat reductions.

**Conclusion:**

Lifestyle modification led to significant reductions in liver fat, and the extent of improvement was influenced by baseline metabolic characteristics. These findings suggest that metabolic profiles, rather than weight loss alone, determine treatment responsiveness and support the use of individualized lifestyle strategies for MASLD management.

## Introduction

Metabolic-associated steatotic liver disease (MASLD) is a prevalent non-communicable disease that poses a significant global health challenge, contributing to both disease-related complications and increased mortality [1–3]. This condition is particularly important because beyond being a liver disease, it is also associated with the heterogeneous metabolic profiles of affected individuals [4–6]. MASLD is diagnosed by the presence of steatotic liver plus at least one of cardiometabolic risk [7–9].

Moreover, monitoring visceral fat by the quantification of liver steatosis is essential for assessing changes in MASLD. The controlled attenuation parameter (CAP) is a non-invasive ultrasound technique used to detect and quantify hepatic steatosis in individuals with MASLD. CAP measures are reportedly substantially correlated with histologically proven liver fat content, rendering it a valuable as non-invasive liver fat assessment in clinical practice [10,11]. CAP is particularly useful for monitoring changes of liver fat following lifestyle modification (LSM).

Previous studies have linked baseline metabolic and anthropometric characteristics with the degree of hepatic steatosis [12–15]; however, whether and to what extent these factors determine the magnitude of liver fat reduction following lifestyle modification in MASLD has not been comprehensively evaluated. This study, therefore, aimed to quantify the relationship between baseline characteristics and changes in CAP after a 6-month behavioral intervention using a telemedicine-based lifestyle program.

## Materials and methods

### Study design and population

This prospective cohort study was conducted between 30 August 2023 and 30 April 2024 and participants were enrolled after they met the screening criteria for MASLD at the endocrine and liver clinic of tertiary hospital. Participants were included if they had hepatic steatosis (CAP ≥ 215 dB/m) [16] with (1) Individuals with diabetes who were receiving medical care or had recently been diagnosed with diabetes, (2) Those who are obese, defined as a BMI ≥ 25.0 kg/m^2^ or central obesity, which is defined as having a waist circumference (WC) of ≥90 cm or ≥80 cm in men and women, respectively. (3) Patients who presented at least 1 of 5 cardiometabolic risk factors. (Blood pressure ≥130/85 mmHg, or on treatment for hypertension; impaired fasting blood sugar (FBS, 100–125 mg/dL); triglyceride level ≥150 mg/dL, or on treatment for dyslipidemia; high-density lipoprotein-C level of ≤39 < 40 and ≤50 < 50 mg/dL in men and women, respectively, or on treatment) The exclusion criteria, which were based on international guidelines [8], included (1) the use of medications known to induce hepatic steatosis, such as corticosteroids, amiodarone, and methotrexate and (2) significant alcohol intake >20 g/d in women and >30 g/d in men. After providing a comprehensive explanation of the research details to the volunteers and ensuring that they understood the research concepts, we enrolled participants who signed the consent form willingly. Each participant underwent a comprehensive history-taking and physical assessment session. Measurements of blood pressure, WC, and baseline weight were recorded. Blood samples were collected for biochemical parameters, and body compositions were evaluated by measuring electrical resistance using bioelectrical impedance analysis. Participants were divided into two groups based on their metabolic baseline.

We calculated the blood-based score using the following variables: (1) The Non-Alcoholic Fatty Liver Disease (NAFLD) Fibrosis Score (NFS) was determined using six variables: age, BMI, aminotransferase ratio, diabetes status, platelet count, and albumin [17,18]; (2) The Fibrosis-4 score (FIB-4) is a prognostic indicator that is used to assess the extent of fibrosis in the liver, considering variables such as age, aminotransferase enzymes, and platelet count [18,19]. Body composition assessment is a technique used to assess the distribution of fat and muscle in the body by measuring the electrical resistance of different tissues [20–22]. The transient elastography device FibroScan® 502 Touch (Echosens, Paris, France), in which ultrasonic technology is used to measure liver stiffness, was used to quantify hepatic steatosis using the CAP value [23]. All measurements were performed using the 3.5 MHz M probe or the 2.5 MHz XL probe as appropriate. The median values of the successful CAP measurements were expressed in decibels per meter (dB/m), whereas the successful liver stiffness values were expressed in kilopascals (kPa). The criterion for dependable CAP measurements comprised at least 10 valid measurements, a success rate of at least 60%, and an interquartile range (IQR) of CAP < 40 dB/m. Steatosis was graded based on the percentage of hepatocytes with fat: ≤ 10% (S0), 11–33% (S1), 34–66% (S2), and >66% (S3) [16].

In these clinics, we recommended daily calorie intake based on the basal metabolic rate (BMR; range: 1200–1500 and 1500–1800 kcal/d for women and men, respectively) and instructed the participants to count calories. We proposed using food samples with reduced calories that are either low in carbohydrates (<130 g/d), high in protein (>1.2 g/d), and low in fat (<20% of total kcal/d). Additionally, we promoted the consumption of vegetables and fiber. The physicians evaluated the food types based on daily photo reports submitted via telemedicine application and during the patient’s interview.

Physical activity levels were classified into three energetic consumption categories based on the World Health Organization and American College of Sports Medicine guidelines. Light activity referred to activities <3.0 metabolic equivalents (METs), moderate activity to 3.0–5.9 METs, and vigorous activity to ≥6.0 METs. Participants engaged in moderately intense physical activity, lasting for a minimum of 150 and 200 min per week for individuals with BMI < 25 and ≥25 kg/m^2^, respectively, for at least 3 days per week. All participants were asked to walk 8000–12,000 steps per day. The target weight reduction for overweight individuals was 3% and participants provided self-reported weight data weekly.

### Ethics approval

The study protocol (COA 144/2566) was approved by the institutional review board of the Faculty of Medicine at Vajira Hospital, and the study was conducted in compliance with the ethical criteria outlined in the Declaration of Helsinki (1975). All participants provided written informed consent before they were recruited. Participants’ information was anonymized and handled confidentially. Only authorized investigators had access to coded data stored in secure institutional databases.

### Statistical analysis

Descriptive statistics were used to outline the variables of the participants. Categorical variables were expressed as frequencies and percentages, whereas continuous variables were represented using the mean and standard deviation (SD), irrespective of their distribution. For the primary objective, multilevel linear (mean difference) regression models with random intercept and random slope by *xtmixed* command in Stata were used to (1) compare the mean difference in CAP at baseline between both groups based on individual characteristics, (2) calculate the mean difference in CAP changes over time for both groups and (3) analyse the effect (slope) of each characteristic on CAP change. A single multivariable multilevel linear regression model was fitted to analyse the mean difference in CAP change over time between the two comparison groups for each characteristic, adjusted for other variables in the model. Before fitting the multilevel linear regression model, we assessed multicollinearity among independent variables using the variance inflation factor (VIF) after a standard linear regression including all covariates. To reduce unnecessary collinearity, non-significant variables that were highly correlated with other covariates but not independently associated with mean CAP change in the univariable analysis were excluded from the final model. Data analysis was conducted using Stata version 18.0 (Stata Corporation, College Station, TX, United States), with statistical significance set at p < 0.05.

## Results

The characteristics of 60 participants enrolled in the study were evaluated. The mean age was 46.93 years, and 70% were female. The average body weight, BMI, and WC were 92.2 ± 23.5 kg, 35.0 ± 7.2 kg/m^2^, and 106.2 ± 16.4 cm, respectively. Of the 60 participants, 27 (45%) were diagnosed with diabetes. The baseline mean FPG was 125.9 ± 40.3 mg/dL, and the glycated hemoglobin (HbA1C) level was 6.7 ± 1.4%. More than half of the cohort had underlying conditions, such as hyperlipidemia and hypertension. Regarding lipid profiles, 28.3% had triglyceride levels of >150 mg/dL and 26.7% had total cholesterol levels of >200 mg/dL. The majority of participants (95%) were obese and exhibited substantial insulin resistance, confirmed with a mean HOMA-IR score of 7.4 ± 5.8. The average percentage of body fat was 42.2 ± 8.0% (Table 1). All participants were confirmed to have fatty liver without elevated liver enzymes. The average CAP was 319.1 ± 42.3 dB/m, and the mean ALT level was 34.4 ± 26.3 IU/L. At baseline, the FIB-4 indicated a low likelihood of liver fibrosis. The mean liver stiffness was within the minimal fibrosis range (7.6 ± 4.7 kPa).

**Table 1:** Characteristics of participants.

Variables	Total (n = 60)
**Endpoint of interest**	
CAP at baseline (dB/m)	319.13 ± 42.33
CAP after 6 months follow-up (dB/m)	292.47 ± 65.45
**Baseline characteristics**	
Liver steatosis grade	
Grade S1	6 (10.0)
Grade S2	13 (21.7)
Grade S3	41 (68.3)
Female	42 (70.0)
Age (years)	46.93 ± 12.16
Body Fat (%)	42.22 ± 8.01
BMI (kg/m^2^)	34.99 ± 7.24
Waist circumference (cm)	106.18 ± 16.38
Hypertension	35 (58.3)
SBP (mm Hg)	134.57 ± 13.55
DBP (mm Hg)	79.78 ± 9.92
Diabetes mellitus	27 (45.0)
Duration of DM (y) (years)	1.96 ± 3.43
FBS (mg/dL)	125.88 ± 40.30
HbA1c (%)	6.66 ± 1.43
HOMA-IR score	7.37 ± 5.79
Total cholesterol (mg/dL)	176.07 ± 41.08
Triglyceride (mg/dL)	141.00 ± 71.52
AST (U/L)	30.02 ± 12.49
ALT (U/L)	34.35 ± 26.25
LS (kPa)	7.57 ± 4.72
NFS score	−0.87 ± 1.45
FIB-4 score	1.02 ± 0.54
eGFR (mL/min/1.73 m^2^)	92.37 ± 22.05
**Dietary and exercise during** **6 months follow-up**	**(n = 52)**
Low total calorie intake/day	15 (28.85)
Low carbohydrate intake/day	39 (75.0)
High protein intake/day	31 (59.6)
Low fat intake/day	4 (7.7)
Step counts ≥8000 steps/day	18 (41.9)
Step counts/day (steps)	7415 ± 2701.47

Data are frequency (%) of participants or mean ± standard deviation.

Abbreviation: ALT, alanine aminotransferase; AST, aspartate aminotransferase; BMI, body mass index; CAP, controlled attenuation parameter; DBP, diastolic blood pressure; DM, diabetes mellitus; eGFR, estimated glomerular filtration rate; FBS, fasting blood sugar;

FIB-4, the fibrosis 4 score; HbA1c, glycated hemoglobin; HOMA-IR, homeostatic model assessment for insulin resistance; LS, liver stiffness; NFS, the non-alcoholic fatty liver disease fibrosis score; SBP, systolic blood pressure.

During the follow-up, 52 participants provided calorie reports; however, only a minority (28.85%) consumed the recommended calories. The majority of participants (75%) followed a low-carbohydrate diet, whereas 59.6% had a high protein intake. Nevertheless, adherence to low fat intake was 7.7%. The physical activity levels varied, with less than average (41.9%) achieving the target of 8000 steps per day, with an average step count of 7415 steps daily.

We explored the effects of various baseline factors by analysing the changes in CAP values from baseline to 6 months (Table 2). In the univariable analysis, females showed a significant decrease in CAP (mean change: −41.81 dB/m, p < 0.001), whereas the increase was non-significant for males (mean change: + 8.67 dB/m, p = 0.492). However, when adjusting for other variables in multivariable analysis, the sex difference in CAP change was no longer significant (p = 0.417).

**Table 2:** Univariable and multivariable mean differences in CAP change over time, estimated by multilevel linear (mean difference) regression models with random intercept and random slope analysis for variables of participants from baseline to 6 months follow-up.

Variables	CAP before (dB/m)	CAP after (dB/m)	Mean CAP change from baseline in two groups (dB/m)	p-value†	Univariable analysis		Multivariable analysis⁋	
					Mean difference of CAP change between two groups (dB/m)	p-value‡	Mean difference of CAP change between two groups (dB/m)	p-value‡
**Baseline characteristics**								
**Sex**	3.05 (−20.12 to 26.21), p 0.797*							
Female	320.05 ± 41.22	278.24 ± 65.19	−41.81 (−57.99 to – 25.63)	<0.001	−50.48 (−80.02 to −20.93)	0.001	−14.05 (−47.99 to 19.89)	0.417
Male	317.00 ± 45.97	325.67 ± 54.30	8.67 (−16.05 to 33.39)	0.492	Ref.		Ref.	
**Age (years)**	−12.42 (−33.87 to 9.04), p 0.257 ‡							
≥ 45	314.17 ± 45.61	271.69 ± 59.81	−42.47 (−60.43 to −24.51)	<0.001	−39.51 (−67.91 to −11.11)	0.006	−44.52 (−84.86 to −4.17)	**0.031**
< 45	326.58 ± 36.54	323.63 ± 62.09	−2.96 (−24.96 to 19.04)	0.792	Ref.		Ref.	
**BMI (kg/m**^**2**^)	−29.26 (−77.43 to 18.91), p 0.234 ‡							
< 25	291.33 ± 39.46	208.00 ± 121.15	−83.33 (−147.64 to −19.03)	0.011	−59.65 (−125.62 to 6.32)	0.076	−78.31 (−139.94 to −16.67)	**0.013**
≥ 25	320.60 ± 42.29	296.91 ± 59.89	−23.68 (−38.44 to −8.93)	0.002	Ref.		Ref.	
**Waist circumference (cm)**	17.16 (−21.02 to 55.35), p 0.378 ‡							
≥ 90 or ≥80	320.56 ± 42.45	292.62 ± 65.95	−27.95 (−43.31 to −12.58)	<0.001	−15.35 (−68.57 to 37.88)	0.572	−87.85 (−153.23 to −22.47)	**0.008**
< 90 or <80	303.40 ± 42.05	290.80 ± 66.74	−12.60 (−63.56 to 38.36)	0.628	Ref.		Ref.	
**Hypertension**	−8.27 (−29.72 to 13.17), p 0.449 ‡							
Yes	315.69 ± 40.11	290.09 ± 69.95	−25.60 (−44.91 to −6.29)	0.009	2.56 (−27.35 to 32.47)	0.867	17.22 (−7.26 to 41.70)	0.168
No	323.96 ± 45.66	295.80 ± 59.82	−28.16 (−51.01 to −5.31)	0.016	Ref.		Ref.	
**Variables**	**CAP before** **(dB/m)**	**CAP after** **(dB/m)**	Mean CAP change from baseline in two groups (dB/m) *	**p-value**†	**Univariable analysis**		**Multivariable analysis**⁋	
					**Mean difference of CAP change between two groups (dB/m)**	**p-value**†	**Mean difference of CAP change between two groups (dB/m)**	**p-value**†
**Diabetes mellitus**	−4.96 (−26.27 to 16.36), p 0.649 ‡							
Yes	316.41 ± 45.17	290.81 ± 59.73	−25.59 (−47.58 to −3.61)	0.023	1.95 (−27.69 to 31.60)	0.897	−61.31 (−100.25 to −22.36)	**0.002**
No	321.36 ± 40.43	293.82 ± 70.67	−27.55 (−47.43 to −7.66)	0.007	Ref.		Ref.	
**Duration of DM (years)**	−29.87 (−57.78 to −7.96), p 0.008 ‡							
< 3	328.10 ± 40.08	291.90 ± 71.44	−36.19 (−53.26 to −19.12)	<0.001	−31.75 (−62.92 to −0.58)	0.046	−47.95 (−83.87 to −12.03)	**0.009**
≥ 3	298.22 ± 41.04	293.78 ± 50.53	−4.44 (−30.52 to 21.63)	0.738	Ref.		Ref.	
**FBS (mg/dL)**	−6.94 (−30.05 to 16.17), p 0.556 ‡							
≤ 100	314.28 ± 48.17	226.33 ± 70.31	−47.94 (−74.09 to −21.79)	<0.001	−30.40 (−61.65 to 0.86)	0.057	−43.49 (−74.00 to −12.99)	**0.005**
> 100	321.21 ± 40.02	303.67 ± 65.45	−17.55 (−34.67 to −0.43)	0.045	Ref.		Ref.	
**HbA1c (%)**	−7.71 (−29.89 to 14.48), p 0.496 ‡							
< 6.5	316.44 ± 41.79	282.69 ± 70.43	−33.74 (−51.79 to −15.70)	<0.001	−20.22 (−50.72 to 10.28)	0.194	Not included	
≥ 6.5	324.14 ± 43.91	310.62 ± 51.77	−13.52 (−38.11 to 11.06)	0.281	Ref.			
**HOMA-IR**	−9.18 (−38.83 to 20.48), p 0.544 ‡							
≤ 2.9	311.33 ± 50.71	232.44 ± 72.87	−78.89 (−114.17 to −43.60)	<0.001	−61.44 (−99.71 to −23.17)	0.002	−17.05 (−52.62 to 18.52)	0.347
> 2.9	320.51 ± 41.11	303.06 ± 58.67	−17.45 (−32.27 to −2.63)	0.021	Ref.		Ref.	
**Total cholesterol (mg/dL)**	18.40 (−5.17 to 41.96), p 0.126 ‡							
> 200	332.63 ± 44.27	296.13 ± 80.29	−36.50 (−64.92 to −8.08)	0.012	−13.41 (−46.59 to 19.77)	0.428	Not included	
≤ 200	314.23 ± 41.02	291.14 ± 60.18	−23.09 (−40.23 to −5.96)	0.008	Ref.			
**Triglyceride (mg/dL)**	4.33 (−19.22 to 27.87), p 0.719 ‡							
> 150	322.24 ± 44.30	291.59 ± 62.25	−30.65 (−58.33 to −2.96)	0.030	−5.55 (−38.26 to 27.15)	0.739	−17.72 (−47.87 to 12.44)	0.250
≤ 150	317.91 ± 42.00	292.81 ± 67.38	−25.09 (−42.50 to −7.69)	0.005	Ref.		Ref.	
**LS (kPa)**	−6.67 (−31.14 to 17.80), p 0.593 ‡							
< 8	317.47 ± 44.72	282.82 ± 63.69	−34.64 (−51.19 to −18.10)	<0.001	−31.91 (−65.00 to 1.18)	0.059	−11.75 (−46.29 to 22.80)	0.505
≥ 8	324.13 ± 35.08	321.40 ± 64.10	−2.73 (−31.39 to 25.93)	0.852	Ref.		Ref.	
**NFS**	3.74 (−18.28 to 25.76), p 0.739 ‡							
< −1.455	321.50 ± 29.05	290.45 ± 58.78	−31.04 (−55.36 to −6.73)	0.012	−6.91 (−37.47 to 23.64)	0.657	−13.38 (−43.33 to 16.58)	0.381
≥ −1.455	317.76 ± 48.72	293.63 ± 69.75	−24.13 (−42.64 to −5.63)	0.011	Ref.		Ref.	
**FIB-4**	−26.91 (−51.08 to −2.74), p 0.029 ‡							
≥ 1.30	298.50 ± 51.26	251.93 ± 64.24	−46.57 (−76.56 to −16.58)	0.002	−25.96 (−60.21 to 8.29)	0.137	1.69 (−36.08 to 39.45)	0.930
< 1.30	325.41 ± 37.65	304.80 ± 61.30	−20.61 (−37.15 to −4.06)	0.015	Ref.		Ref.	
**eGFR (mL/min/1.73 m**^**2**^)	−20.53 (−53.20 to 12.15), p 0.218 ‡							
≤ 59	301.00 ± 56.57	281.86 ± 77.67	−19.14 (−62.28 to 23.99)	0.384	8.52 (−37.38 to 54.41)	0.716	−19.17 (−79.83 to 41.48)	0.535
> 59	321.53 ± 40.17	293.87 ± 64.40	−27.66 (−43.34 to −11.98)	0.001	Ref.		Ref.	
**Characteristics during telemedicine-based lifestyle modification program**								
**Low total calories intake/day**	−10.38 (−35.26 to 14.50), p 0.413 ‡							
Yes	326.87 ± 45.73	299.13 ± 53.82	−27.73 (−58.26 to 2.79)	0.075	−3.95 (−40.14 to 32.24)	0.831	−12.72 (−56.65 to 31.20)	0.570
No	316.49 ± 40.87	292.70 ± 73.84	−23.78 (−43.22 to −4.35)	0.016	Ref.		Ref.	
**Low carbohydrate intake/day**	14.13 (−11.78 to 40.04), p 0.285 ‡							
Yes	330.08 ± 36.31	289.85 ± 61.50	−40.23 (−72.68 to −7.78)	0.015	−20.41 (−57.88 to 17.06)	0.286	−41.97 (−76.81 to −7.13)	**0.018**
No	315.95 ± 43.76	296.13 ± 70.99	−19.82 (−38.56 to −1.08)	0.038	Ref.		Ref.	
**High protein intake/day**	18.62 (−3.94 to 41.17), p 0.106 ‡							
Yes	327.00 ± 39.09	288.16 ± 76.52	−38.84 (−59.23 to −18.44)	<0.001	−34.46 (−66.55 to −2.37)	0.035	−31.51 (−61.50 to −1.53)	**0.039**
No	308. 38 ± 44.93	304.00 ± 54.08	−4.38 (−29.16 to 20.40)	0.729	Ref.		Ref.	
**Low fat intake/day**	20.56 (−21.63 to 62.76), p 0.340 ‡							
Yes	300.50 ± 46.87	313.00 ± 79.64	12.50 (−45.69 to 70.69)	0.674	40.54 (−20.02 to 101.10)	0.190	29.28 (−47.36 to 105.92)	0.454
No	321.06 ± 41.87	293.02 ± 67.92	−28.04 (−44.84 to −11.24)	0.001	Ref.		Ref.	
**Step counts/day (steps)**	−5.38 (−29.56 to 18.80), p 0.663 ‡							
≥ 8000	312.22 ± 47.68	268.00 ± 75.30	−44.22 (−70.79 to −17.66)	0.001	−24.98 (−59.82 to 9.86)	0.160	−16.94 (−44.76 to 10.89)	0.233
< 8000	317.60 ± 35.27	298.36 ± 53.82	−19.24 (−41.78 to 3.30)	0.094	Ref.		Ref.	

Data are mean ± standard deviation or mean difference (95%Confidence interval), significant level were set at p < 0.05.

* Multilevel linear (mean difference) regression models with random intercept and random slope were used to analyse mean difference in CAP at baseline between two comparison groups.

† Multilevel linear (mean difference) regression models with random intercept and random slope were used to analyse mean difference in CAP from baseline to 6 months both comparison groups.

‡ Multilevel linear (mean difference) regression models with random intercept and random slope were used to analyse mean difference in CAP change over time (slope) between two comparison groups.

⁋ Multivariable analysis adjusted for sex, age, BMI, waist circumference, hypertension, diabetes mellitus, duration of DM, FBS, HOMA-IR, triglyceride, LS, NFS, FIB-4, eGFR, total calories intake/day, carbohydrate intake/day, protein intake/day, fat intake/day, step counts/day.

Abbreviation: BMI, body mass index; CAP, controlled attenuation parameter; DM, diabetes mellitus; eGFR, estimated glomerular filtration rate; FBS, fasting blood sugar; FIB-4, the fibrosis 4 score; HbA1c, glycated hemoglobin; HOMA-IR, homeostatic model assessment for insulin resistance; LS, liver stiffness; NFS, the non-alcoholic fatty liver disease fibrosis score.

Participants aged ≥ 45 years experienced a significant reduction in CAP (mean change: -42.47 dB/m, p < 0.001) compared with those aged < 45 years, who had minimal change (mean change: −2.96 dB/m, p = 0.792). From the multivariable analysis, we confirmed that older participants exhibited significantly more reduced CAP than their younger counterparts (mean difference: −44.52 dB/m, p = 0.031).

**Fig 1:**
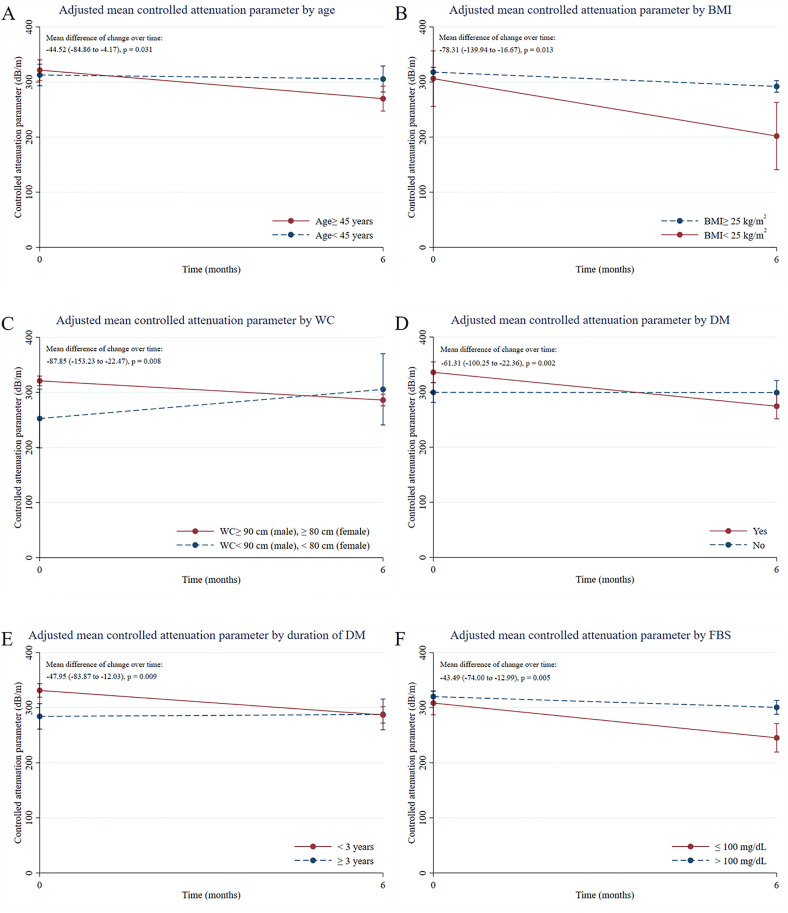
Adjusted mean controlled attenuation parameter change over time based on significant baseline characteristics: A, age; B, body mass index (BMI); C, waist circumference (WC); D, diabetes mellitus (DM); E, duration of DM; F, fasting blood sugar (FBS).

Participants with a BMI ≥ 25 kg/m^2^ showed a lower reduction in CAP than those with a BMI < 25 kg/m^2^. In the univariable analysis, the mean CAP change in the BMI ≥ 25 group was −23.68 dB/m (p = 0.002), whereas another group showed a much larger reduction of −83.33 dB/m (p = 0.011). After adjusting for other factors, individuals with BMI < 25 kg/m^2^ experienced a significantly greater reduction in CAP than those in other groups (mean difference: −78.31 dB/m, p = 0.013) (Table 2, Fig 1).

Participants with a larger WC had significantly reduced CAP values (mean change: −27.95 dB/m, p < 0.001), while those with smaller WC showed a non-significant change (−12.60 dB/m, p = 0.628). Multivariable analysis revealed that a larger WC was associated with a significantly greater reduction in CAP (mean difference: −87.85 dB/m, p = 0.008) (Table 2, Fig 1).

In the univariable analysis, participants with diabetes showed a significant reduction in CAP values (−25.59 dB/m, p = 0.023), with similar reductions observed for those without diabetes (−27.55 dB/m, p = 0.007). However, in multivariable analysis, those with diabetes mellitus showed a more substantial reduction in CAP (mean difference: - 61.31dB/m, p = 0.002) (Table 2, Fig 1).

In the univariable analysis, participants with FBS ≤ 100 mg/dL had significantly reduced CAP values (−47.94 dB/m, p < 0.001), whereas those with FBS > 100 mg/dL showed lower reductions (−17.55 dB/m, p = 0.045). In the multivariable analysis, the difference in CAP reduction between the two FBS groups remained significant (mean difference: −43.49 dB/m, p = 0.005) (Table 2, Fig 1).

The analysis revealed that certain factors, such as lipid profile and all the non-invasive tests for liver fibrosis (FIB-4, NFS, liver stiffness), were significantly associated with CAP changes in the univariable analysis; however, these effects were not maintained after adjusting for other variables in the multivariable model.

Dietary and physical activity factors had varying effects on the liver fat content. Even though participants followed the dietary recommended value (1500 kcal/d or 1200 kcal/d), a non-significant CAP reduction of −27.73 dB/m (p = 0.075) was achieved. Moreover, after adjusting for potential confounders, the mean difference in CAP change was not significant (p = 0.570).

Participants with a low carbohydrate intake had a significant CAP reduction of −40.23 dB/m (p = 0.015) and this reduction remained significant at (−41.97 dB/m, p = 0.018). High protein intake was associated with a substantial CAP reduction of −38.84 dB/m (p < 0.001). The multivariable analysis revealed a significant CAP reduction of −31.51 dB/m (p = 0.039). For low-fat intake, the mean difference of CAP change was not statistically significant.

Participants with increased daily step counts (≥8000 steps) showed a significant CAP reduction of −44.22 dB/m (p = 0.001). However, after adjusting for confounders, this effect was not significant (mean difference of −16.94 dB/m, p = 0.233).

## Discussion

In a previous study, patients with chronic liver disease who were not diabetic were reported to have reduced hepatic steatosis after adhering to a well-balanced diet and engaging in regular exercise for 6 months [24]. A decrease in CAP is positively correlated with a reduction in weight and BMI; however, it is not correlated with age, insulin resistance, hepatic inflammation, or cholesterol [24]. We investigated the details of both baseline characteristics and LSM, grouping each parameter according to severity to clarify the impact of individual parameters on both the dynamic and magnitude of the difference in CAP change in participants with MASLD. Over the 6-month follow-up period, our findings revealed that baseline independent characteristics, including older age, high WC, and a reduced BMI, were associated with greater reductions in CAP values. In this study, we emphasized the significance of each independent factor as a potentially important contributor to the successful reduction of CAP. These characteristics may be crucial data for the multidisciplinary team to encourage patients to adhere to LSM.

Interestingly, participants aged ≥45 years showed greater reductions in CAP compared with younger individuals. This finding suggests that age may influence the metabolic responsiveness to LSM. Older participants might demonstrate better adherence to behavioral interventions and dietary recommendations, resulting in more consistent lifestyle changes. These results collectively imply that age-related metabolic adaptation rather than chronological aging itself may contribute to greater hepatic fat reduction in older individuals.

In this study, a BMI cutoff of ≥ 25 kg/m^2^ was applied in accordance with WHO and Asian population criteria to indicate increased adiposity and metabolic risk [25,26]. This threshold allowed inclusion of participants with truncal obesity despite having lower BMI values, thereby capturing individuals at risk for MASLD. The observed greater reduction in CAP among those with lower BMI but higher waist circumference supports the notion that visceral adiposity, rather than overall body weight, plays a more critical role in predicting hepatic fat improvement following LSM.

We emphasized in this study that individuals with diabetes, particularly those with improved glycemic control and shortened disease duration, experienced more substantial reductions in liver fat. The mitochondrial dysfunction and decreased insulin sensitivity in the visceral adipose tissue is a main mechanism of de novo lipogenesis which promotes liver fat accumulation in diabetes [27,28]. In this context, other markers, such as insulin resistance and HbA1c levels, may not have a direct impact. After adjusting for potential confounders, the nonsignificant difference in mean CAP change between the degree of insulin resistance was caused by individuals with pre-existing, well-controlled diabetes and the brief duration of lifestyle monitoring.

In a previous study of participants with steatohepatitis, the dynamic change in CAP and liver stiffness was not significantly associated [24]. The benefits of the dynamic CAP difference after LSM and the stratified initial degree of non-invasive liver fibrosis were not identified in our study, as participants did not have advanced liver fibrosis or liver inflammation at baseline. All participants in this study had fatty liver without elevated liver enzymes, suggesting simple steatosis rather than steatohepatitis. Since liver enzyme levels were within the normal range, subgroup analysis based on these parameters was not conducted.

Maintaining telehealth communication, whether online or face-to-face, facilitates interaction between healthcare providers and patients across multiple dimensions. Previous studies that highlighted histological steatosis improvement with a 1-year LSM, reported a desired outcome of a weight loss of at least 5% [29] and 7% [30]. Early target weight reduction was achieved by the majority of our participants. Our study presents findings on the beneficial effects of liver fat reduction upon the attainment of a 3% weight loss, which was achieved through individuals’ LSM.

A low-carbohydrate diet reduces hepatic steatosis by decreasing insulin levels, enhancing lipolysis and fat oxidation, and diminishing de novo lipogenesis [31]. In previous studies, the relationship of a low-carbohydrate diet with either diminished CAP [32] or a dynamic drop in CAP was reported [33]. Previous meta-analyses revealed that both low-carbohydrate and low-fat diet groups had improved hepatic fat; nonetheless, the effect of the two dietary strategies did not significantly differ [34]. Our results did not show the advantages of either low-fat or low-calorie diets because the number of self-reports was insufficient. A high-protein diet can ameliorate liver fat by increasing satiety and lowering calorie intake, enhancing insulin sensitivity, and inhibiting fat absorption and lipid synthesis [35]. Previous studies reported an association between a low-protein diet and the occurrence of hepatic steatosis [36]. Our findings show that a high protein intake is related to a significantly dynamic reduction in CAP, hence supporting the conceptual mechanism.

Despite the increased daily step counts initially linked to improved liver fat content, this relationship was not sustained after adjusting for confounding variables. This suggests that other factors, such as the type of exercise and duration of LSM, may affect the relationship between physical activity and liver fat levels. Our expertise facilitated the prioritization of MASLD management, especially in lean MASLD, truncal obesity, and diabetes, to ensure effective CAB modulation, particularly in resource-constrained scenarios.

The main strengths of this study include the quantitative evaluation the magnitude of liver fat reduction using the CAP, providing an objective and non-invasive measure of metabolic improvement. Subgroup analyses allowed identification of differential responses to LSM.

The study had some limitations. The statistical power and generalizability of the findings were limited owing to the small sample size in our single-center study. For more robust conclusions, a larger cohort is required. Additionally, dietary intake and physical activity data were self-reported, introducing potential bias. The 6-month follow-up period is relatively short to assess the long-term effects of LSM on liver fat reduction. Hence, adopting a prolonged follow-up would provide insights into the sustainability of these findings. Finally, genetic predisposition and polymorphisms known to influence hepatic fat accumulation were not assessed in this study. Consequently, a greater number of participants with MASLD, particularly those with lean diabetes, is necessary for future research.

## Conclusions

This study demonstrated that baseline metabolic characteristics significantly influence the reduction of liver fat measured by CAP after six months of lifestyle modification. Older age, higher waist circumference, lower BMI, and better glycemic control were associated with greater CAP reduction, indicating that metabolic profiles rather than body weight alone determine responsiveness to behavioral interventions. These findings highlight the potential of individualized lifestyle strategies—particularly for diabetic and lean MASLD patients—to optimize non-pharmacologic management of hepatic steatosis.

## Supporting information

S1 File Confirm data availability statement.
(DOCX)

S2 File Minimal data set.
(XLSX)

## References

[1] Devarbhavi H, Asrani SK, Arab JP, Nartey YA, Pose E, Kamath PS. Global burden of liver disease: 2023 update. J Hepatol. 2023;79(2):516–37. doi: 10.1016/j.jhep.2023.03.017 36990226

[2] Le P, Tatar M, Dasarathy S, Alkhouri N, Herman WH, Taksler GB, et al. Estimated Burden of Metabolic Dysfunction-Associated Steatotic Liver Disease in US Adults, 2020 to 2050. JAMA Netw Open. 2025;8(1):e2454707. doi: 10.1001/jamanetworkopen.2024.54707 39821400 PMC11742522

[3] Sethasine S, Simasingha N, Ratana-Amornpin S, Mahachai V. Real world for management of hepatocellular carcinoma: a large population-based study. Scand J Gastroenterol. 2023;58(10):1153–8. doi: 10.1080/00365521.2023.2209686 37203205

[4] Phaloprakarn C, Sethasine S. Association between glucose intolerance and fatty liver disease in women with previous gestational diabetes mellitus in urban Thailand: a prospective cohort study. BMJ Open. 2025;15(5):e097114. doi: 10.1136/bmjopen-2024-097114 40335146 PMC12056645

[5] Sethasine S, Phaloprakarn C. Relationship between breastfeeding and hepatic steatosis in women with previous gestational diabetes mellitus. Int Breastfeed J. 2024;19(1):75. doi: 10.1186/s13006-024-00684-3 39533322 PMC11555891

[6] Sethasine S, Suthasmalee S, Tangjitgamol S, Phaloprakarn C. Contraception and nonalcoholic fatty liver disease in women with prior gestational diabetes mellitus. Contraception. 2025;145:110860. doi: 10.1016/j.contraception.2025.110860 40021110

[7] Yanai H, Adachi H, Hakoshima M, Iida S, Katsuyama H. Metabolic-Dysfunction-Associated Steatotic Liver Disease-Its Pathophysiology, Association with Atherosclerosis and Cardiovascular Disease, and Treatments. Int J Mol Sci. 2023;24(20):15473. doi: 10.3390/ijms242015473 37895151 PMC10607514

[8] European Association for the Study of the Liver (EASL), European Association for the Study of Diabetes (EASD), European Association for the Study of Obesity (EASO). EASL-EASD-EASO Clinical Practice Guidelines on the management of metabolic dysfunction-associated steatotic liver disease (MASLD). J Hepatol. 2024;81(3):492–542. doi: 10.1016/j.jhep.2024.04.031 38851997

[9] Eslam M, Sarin SK, Wong VW-S, Fan J-G, Kawaguchi T, Ahn SH, et al. The Asian Pacific Association for the Study of the Liver clinical practice guidelines for the diagnosis and management of metabolic associated fatty liver disease. Hepatol Int. 2020;14(6):889–919. doi: 10.1007/s12072-020-10094-2 33006093

[10] Chan W-K, Nik Mustapha NR, Mahadeva S. Controlled attenuation parameter for the detection and quantification of hepatic steatosis in nonalcoholic fatty liver disease. J Gastroenterol Hepatol. 2014;29(7):1470–6. doi: 10.1111/jgh.12557 24548002

[11] Pu K, Wang Y, Bai S, Wei H, Zhou Y, Fan J, et al. Diagnostic accuracy of controlled attenuation parameter (CAP) as a non-invasive test for steatosis in suspected non-alcoholic fatty liver disease: a systematic review and meta-analysis. BMC Gastroenterol. 2019;19(1):51. doi: 10.1186/s12876-019-0961-9 30961539 PMC6454693

[12] Arita VA, Cabezas MC, Hernández Vargas JA, Trujillo-Cáceres SJ, Mendez Pernicone N, Bridge LA, et al. Effects of Mediterranean diet, exercise, and their combination on body composition and liver outcomes in metabolic dysfunction-associated steatotic liver disease: a systematic review and meta-analysis of randomized controlled trials. BMC Med. 2025;23(1):502. doi: 10.1186/s12916-025-04320-7 40866968 PMC12392582

[13] Yeo YH, Zhu Y, Gao J, Liu S, Ni W, Rui F, et al. Anthropometric Measures and Mortality Risk in Individuals With Metabolic Dysfunction-Associated Steatotic Liver Disease (MASLD): A Population-Based Cohort Study. Aliment Pharmacol Ther. 2025;62(2):168–79. doi: 10.1111/apt.70174 40366297

[14] Krönert N, Moulla Y, Lange UG, Blüher M, Linder N, Fuhrmann A, et al. A hypocaloric protein-rich diet before metabolic surgery improves liver function in patients with obesity and diabetes : A secondary analysis of a randomized clinical trial. Langenbecks Arch Surg. 2025;410(1):36. doi: 10.1007/s00423-024-03600-9 39804512 PMC11729132

[15] Askeland-Gjerde DE, Westlye LT, Andersson P, Korbmacher M, de Lange A-M, van der Meer D, et al. Mediation Analyses Link Cardiometabolic Factors and Liver Fat With White Matter Hyperintensities and Cognitive Performance: A UK Biobank Study. Biol Psychiatry Glob Open Sci. 2025;5(4):100488. doi: 10.1016/j.bpsgos.2025.100488 40330223 PMC12052680

[16] de Lédinghen V, Vergniol J, Foucher J, Merrouche W, le Bail B. Non-invasive diagnosis of liver steatosis using controlled attenuation parameter (CAP) and transient elastography. Liver Int. 2012;32(6):911–8. doi: 10.1111/j.1478-3231.2012.02820.x 22672642

[17] Angulo P, Hui JM, Marchesini G, Bugianesi E, George J, Farrell GC, et al. The NAFLD fibrosis score: a noninvasive system that identifies liver fibrosis in patients with NAFLD. Hepatology. 2007;45(4):846–54. doi: 10.1002/hep.21496 17393509

[18] European Association for the Study of the Liver. EASL Clinical Practice Guidelines on non-invasive tests for evaluation of liver disease severity and prognosis - 2021 update. J Hepatol. 2021;75(3):659–89. doi: 10.1016/j.jhep.2021.05.025 34166721

[19] Lee J, Vali Y, Boursier J, Spijker R, Anstee QM, Bossuyt PM, et al. Prognostic accuracy of FIB-4, NAFLD fibrosis score and APRI for NAFLD-related events: A systematic review. Liver Int. 2021;41(2):261–70. doi: 10.1111/liv.14669 32946642 PMC7898346

[20] Jackson AS, Pollock ML. Practical Assessment of Body Composition. Phys Sportsmed. 1985;13(5):76–90. doi: 10.1080/00913847.1985.11708790 27463295

[21] Segal KR, Gutin B, Presta E, Wang J, Van Itallie TB. Estimation of human body composition by electrical impedance methods: a comparative study. J Appl Physiol (1985). 1985;58(5):1565–71. doi: 10.1152/jappl.1985.58.5.1565 3997721

[22] Lemos T, Gallagher D. Current body composition measurement techniques. Curr Opin Endocrinol Diabetes Obes. 2017;24(5):310–4. doi: 10.1097/MED.0000000000000360 28696961 PMC5771660

[23] Wong VW-S, Vergniol J, Wong GL-H, Foucher J, Chan HL-Y, Le Bail B, et al. Diagnosis of fibrosis and cirrhosis using liver stiffness measurement in nonalcoholic fatty liver disease. Hepatology. 2010;51(2):454–62. doi: 10.1002/hep.23312 20101745

[24] Paul J, Venugopal RV, Peter L, Shetty KNK, Shetti MP. Measurement of controlled attenuation parameter: a surrogate marker of hepatic steatosis in patients of nonalcoholic fatty liver disease on lifestyle modification - a prospective follow-up study. Arq Gastroenterol. 2018;55(1):7–13. doi: 10.1590/S0004-2803.201800000-07 29561981

[25] WHO Expert Consultation. Appropriate body-mass index for Asian populations and its implications for policy and intervention strategies. Lancet. 2004;363(9403):157–63. doi: 10.1016/S0140-6736(03)15268-3 14726171

[26] Haam J-H, Kim BT, Kim EM, Kwon H, Kang J-H, Park JH, et al. Diagnosis of Obesity: 2022 Update of Clinical Practice Guidelines for Obesity by the Korean Society for the Study of Obesity. J Obes Metab Syndr. 2023;32(2):121–9. doi: 10.7570/jomes23031 37386771 PMC10327686

[27] Gancheva S, Roden M, Castera L. Diabetes as a risk factor for MASH progression. Diabetes Res Clin Pract. 2024;217:111846. doi: 10.1016/j.diabres.2024.111846 39245423

[28] Frankowski R, Kobierecki M, Wittczak A, Różycka-Kosmalska M, Pietras T, Sipowicz K, et al. Type 2 Diabetes Mellitus, Non-Alcoholic Fatty Liver Disease, and Metabolic Repercussions: The Vicious Cycle and Its Interplay with Inflammation. Int J Mol Sci. 2023;24(11):9677. doi: 10.3390/ijms24119677 37298632 PMC10254034

[29] Vilar-Gomez E, Martinez-Perez Y, Calzadilla-Bertot L, Torres-Gonzalez A, Gra-Oramas B, Gonzalez-Fabian L, et al. Weight Loss Through Lifestyle Modification Significantly Reduces Features of Nonalcoholic Steatohepatitis. Gastroenterology. 2015;149(2):367-78.e5; quiz e14-5. doi: 10.1053/j.gastro.2015.04.005 25865049

[30] Promrat K, Kleiner DE, Niemeier HM, Jackvony E, Kearns M, Wands JR, et al. Randomized controlled trial testing the effects of weight loss on nonalcoholic steatohepatitis. Hepatology. 2010;51(1):121–9. doi: 10.1002/hep.23276 19827166 PMC2799538

[31] Mardinoglu A, Wu H, Bjornson E, Zhang C, Hakkarainen A, Räsänen SM, et al. An Integrated Understanding of the Rapid Metabolic Benefits of a Carbohydrate-Restricted Diet on Hepatic Steatosis in Humans. Cell Metab. 2018;27(3):559-571.e5. doi: 10.1016/j.cmet.2018.01.005 29456073 PMC6706084

[32] Li X, Li M, Xu L, Zeng X, Zhang T, Yang H, et al. Associations between low-carbohydrate and low-fat diets and hepatic steatosis. Obesity (Silver Spring). 2022;30(11):2317–28. doi: 10.1002/oby.23551 36058841

[33] Chen J, Huang Y, Xie H, Bai H, Lin G, Dong Y, et al. Impact of a low-carbohydrate and high-fiber diet on nonalcoholic fatty liver disease. Asia Pac J Clin Nutr. 2020;29(3):483–90. doi: 10.6133/apjcn.202009_29(3).0006 32990607

[34] Ahn J, Jun DW, Lee HY, Moon JH. Critical appraisal for low-carbohydrate diet in nonalcoholic fatty liver disease: Review and meta-analyses. Clin Nutr. 2019;38(5):2023–30. doi: 10.1016/j.clnu.2018.09.022 30314924

[35] Xu C, Markova M, Seebeck N, Loft A, Hornemann S, Gantert T, et al. High-protein diet more effectively reduces hepatic fat than low-protein diet despite lower autophagy and FGF21 levels. Liver Int. 2020;40(12):2982–97. doi: 10.1111/liv.14596 32652799

[36] Charatcharoenwitthaya P, Tansakul E, Chaiyasoot K, Bandidniyamanon W, Charatcharoenwitthaya N. Dietary Composition and Its Association with Newly Diagnosed Nonalcoholic Fatty Liver Disease and Insulin Resistance. Nutrients. 2021;13(12):4438. doi: 10.3390/nu13124438 34959990 PMC8708546

