# Supplementary material for: Factors influencing effective decrease of controlled attenuation parameters in metabolic-associated steatotic liver disease: A multilevel linear regression analysis at Vajira Hospital
Source: PLoS One. 2025 Dec 3;20(12):e0336294. doi: 10.1371/journal.pone.0336294 (PMC12674518; doi:10.1371/journal.pone.0336294)
Supplement: S1 File — Confirm data availability statement. (DOCX) [file pone.0336294.s001.docx]

28/10/68

Dear editor

Our Data Availability statement as follows:

"All relevant data are within the manuscript and its Supporting Information files."

We confirmed that this statement is wholly accurate and that all intended Supporting Information files have been uploaded.

Supatsri Sethasine

Corresponding author
